# Applied Study of the Fluidization Model of Logistics Transportation through the Prism of the Impact Generated on the Environment

**DOI:** 10.3390/s22239255

**Published:** 2022-11-28

**Authors:** Eduard Zadobrischi, Mihai Negru

**Affiliations:** 1 Department of Computers, Electronics and Automation, Faculty of Electrical Engineering and Computer Science, “Stefan cel Mare” University, No.13. Str. Universitatii, 720229 Suceava, Romania; 2 Department of Computer Science, Technical University of Cluj-Napoca, Gh. Baritiu St. 26-28, 400027 Cluj-Napoca, Romania

**Keywords:** traffic modelling, freight transport, intelligent system transportation model, traffic congestion, critical transportation infrastructure, polluting emissions

## Abstract

A real problem of today’s society is the loss of human lives due to road accidents and the pollution caused by freight transport through metropolitan areas. The restrictions imposed in the near future for freight transport could reduce its efficiency and create many more problems. Using data centralization and developing applications or algorithms dedicated to the freight transportation sectors, routes and emissions can be managed much more efficiently. In this work, general aspects are presented, as well as a route optimization model for freight transport, taking into account the environmental impact, based on a heuristic algorithm, that of the ant colony (ACO). A multitude of studies has focused on what represents the benefits created by the applicability of solutions rather than on generalities and perspectives. The paper aims to highlight the usefulness of an optimization model of freight transport routes and the minimization of time and social costs. The study will show us that an optimized route for freight transport has a huge impact on costs, but also on time efficiency and polluting emissions.

## 1. Introduction

The technological advance and the rapid expansion of the economy, but also the increase in the density of urban populations in Romania have generated problems such as traffic congestion, road accidents, and environmental pollution, which have become extremely disturbing aspects, affecting the social life and the sustainable development of the cities [1]. One of the factors that negatively contribute to the problems listed above is freight transportation. To solve these problems, the traffic management departments tried to make changes and limit the routes that oversize vehicles can follow, especially those with emissions, the goal is to avoid traffic congestion but also to reduce emissions. The generation of new transport policies through which legislative regulations, fines, and restrictions were imposed in certain areas in view of reducing carbon emissions, all these solutions did not provide the expected results. Some new directions relate to the implementation of communication systems based on 5 G in direct collaboration between Ericsson and Einride [2], through which the companies managed to coordinate autonomous trucks on the Goteborg—Barcelona route without polluting, everything being coordinated from a distance, finding the most suitable routes. These approaches generate the expected result, but in terms of costs, they are extremely difficult to bear. However, in terms of the current state, there are still extremely many deficiencies between the impact created by freight transportation and the benefits it brings to the logistics processes. Thus, we can say that there is no definitive standard regarding urban logistics policies that favor solving problems of a congestive nature or pollution in urban environments. The authors of [3] offered a first series of conclusions following the experimental simulation evaluations through which the combined measurements based on the restrictions imposed on oversized transport and the costs that limit transit generated a positive effect by improving emissions in urban areas. Instead, the authors of [4] proposed increasing the full charging rate in order to generate some emission reductions in polluted areas. Thus, aspects are supported through which the use of ecological vehicles becomes an extremely good solution in choosing a sustainable urban development direction in some cities [5]. Although there were studies on the benefits of implementing a policy to penalize gas emissions emitted by trucks carrying out freight transport [6], the results showed that this policy did not come with a viable solution, and emissions continued to increase beyond the initial limit, and the urbanization process of the respective areas was in a continuous expansion. We can say that some of the traffic characteristics of the sub-urban arteries have changed radically, thanks to these changes, those arteries supported much more intense traffic, and at the moment they are turning into alternative routes for extremely short distances because of the density of vehicles. This density has created a new phenomenon, called mixed traffic, in which other forms of transit are also found, from various vehicles to pedestrians, an aspect that causes extremely many accidents resulting in the loss of human lives, high noise, air pollution, and reduced impact economic and the progress of the respective area. Many departments worldwide implement traffic control measures with blocking zones and one-way traffic, strictly dedicated to freight transport in order to exclude the static perspective, dynamizing and fluidizing congested areas. There is not so much research to support some of the proposals, especially since those areas with restrictions in some cases involve costs and increases in public transport, which dramatically reduce the standard of living and the comfort of society. The most common solutions come down to the aspects regarding the restriction and division into transit zones or city bypass belts, all of which aim to minimize traffic congestion, but also environmental pollution. These solutions solve some of the problems, but on the other hand lead to a reduction in the efficiency of freight transport, generating much higher costs for service providers [7,8]. On the other hand, some research lacks detailed and quantitative analyses, empirical methods are used in the development of those traffic management policies.

Many of the studies are based on the modeling and simulation of data that come only from one of the parties under study, in the respective cases of the entrepreneurs. Thus, many of the inflexibilities generated by policies are distorted because of this. Highlighting the presented elements, we try to test and implement a method to optimize the route of a freight transport vehicle with the main goal of minimizing transport and social costs in addition to carbon emissions. Within those social costs are included the costs of traffic congestion, emissions, and the safety of traffic participants, but also of pedestrians. The research requires the presentation of solutions to increase the freight transport dynamics and the implementation at the level of intelligent transport systems (ITS) [9]. In addition to these aspects, the inclusion of cargo transport vehicles in a vehicle network and a data connection through Big Data or IoT can be an extremely easy solution in the real-time transportation of trucks on alternative routes. The structure of article is organized as follows. In Section 2, the relevant approaches and references from specialized literature, traffic models, and analyzes regarding the management of problems generated by freight transport are analyzed. Section 3 proposes simulation models applied to the real infrastructure that must highlight the efficiency created at the level of decision-making factors. In Section 4, the presented aspects, the results, and the effects generated at the level of simulations are analyzed, as also the presentation of the simulation models with practical results, the case study, and the final implementation. In Section 5, the general conclusions and the most important aspects to which the research carried out in the article led, as well as future directions, are presented.

## 2. Discussion Regarding Urban Transport Characteristics and the Evolution of Urban Densities

In order to reduce the negative problems created by freight transport, many of the departments dedicated to traffic management, implement all kinds of solutions and take measures with the aim of controlling the traffic in order to fluidize it and provide free paths for trucks transiting urban areas. The most common measure is the total restriction of trucks in transit areas, this measure can significantly reduce the flow of traffic in those areas or on adjacent routes, but later other problems are caused [10]. These restrictions, over time, will cause delays in freight transport and will form waiting queues, seriously affecting the quality of distribution and goods delivery services [11,12]. An extremely supported example is that many transport companies use passenger vehicles in the process of moving core goods to reduce transit costs, an aspect that automatically increases traffic density and environmental pollution in those areas [13]. We can say that part of the current policies and strategies or applied models or algorithms used in the related problems generated by the studied subject are treated in this chapter.

### 2.1. Presentation of Approaches Regarding Transport Policies and Imposed Limitations

According to studies and journals published over time, aspects regarding transport policies and how punishments or incentives have affected the behavior of car drivers are explained. If we talk about urban transport policies, a large part of the cities adopts punitive policies, such as sanctions for trucks that enter prohibited areas or for exceeding the gauge. One of the main goals of the implementation of urban penalty policies for oversize vehicles is the alleviation of urban traffic congestion and the reduction of pollution. We can say that part of the urban traffic congestion and environmental pollution is largely caused by this branch of transport. According to statistics, any delay or loss of carriageways can lead to congestion, which, in turn, generates costs of over 1–3% of the total GDP of the countries in the European Union [14]. Right from the beginning of the century, one of the examples in Rome, which has an area of over 5–6 square kilometers, and the central area is designated as being restricted to heavy tonnage traffic. This case is also found in other big European cities, such as Paris, Prague, and Oslo [15]. There are solutions implemented in warm areas where, during summer, traffic is restricted by up to 50% for vehicles that do not have a catalyst for filtering noxious, these being prohibited in urban areas [16]. In one of the most developed countries in the European Union, more precisely, Germany, following consultations between the governments and the departments dedicated to the urban planning and traffic sector, a series of restrictions were agreed upon to limit trucks that exceed a certain limit of age, more precisely those over 10 years old. In the case of highways, taxes have been implemented for trucks that exceed the loading volume by more than 10 tons [17]. In the case of countries in the East, traffic time intervals and controls have been established to reduce the density of vehicles on public roads. In the case of countries in Asia, such as those China, registration numbers were implemented in an even-odd system with limitations regarding the transit regime of the zones, succeeding that in some areas such as Shanghai, Tianjin, or Guangzhou, the traffic and its density be reduced by the set of imposed measures.

### 2.2. Studies and Analyzes Regarding the Restriction of Freight Transport and Methods

The most important studies have focused on the analysis and restriction of freight transport because it is a major factor in pollution and the number of emissions it generates. Therefore, the study of the emission model is aimed at evaluating the efficiency and generating restriction protocols for high-tonnage transport. Research teams had as their objective the quantitative evaluation of emissions, and depending on the level of application, they were divided into three categories: macro, medium, and micro [18]. In the case of the macroscopic evaluation for pollutant emissions, emission factors are used that are based on the average speed as the main parameter in their evolutionary calculation. The macro-emissions models are based on mobile, EMFAC, or COPERT models. When we talk about average models, they are used to analyze emissions for each lane or for certain areas, they are based on a scale that includes micro or measurement-type models. Therefore, we can say that the micro model can only analyze emissions from certain areas, roads, or intersections, but the most important assessment is in real-time [19].

According to the studies carried out, in 1959, the Dutch computer scientist Dijkstra proposed the following algorithm named after his own name [20]. It started from the main idea by which a path tree is built by increasing the length of the path but based on the nodes generated between the leaves of the tree and the respective path, obtaining the shortest route from the root node to the node of leaf type. The research did not end, and in 1962, the researcher Floyd proposed the algorithm of the same name [21], which constitutes the shortest matrix of a road by drawing the distance between every two points within a graph passing through the weight matrix of the graph. Even if they appear to be treated after a lot of research, these are classic optimization algorithms for running paths and solving problems of this kind through precise algorithms such as heuristics. In conditions where the size of a problem is small, algorithms can have an optimal contribution in terms of solving in a relatively acceptable time, in this directive, there is research on VRP precision or the cascade representation of decision matrices from a tree dedicated to the number of interruptions in the initial set [22]. Thus, in this article, aspects related to the routing analyzes of some freight transport vehicles will be highlighted in which the impact on the environment is treated. Starting from the aspect related to the analysis of problems for classical routing methods, heuristic algorithms but also standard algorithms such as the bee swarm, the ant colony algorithm, or parallel searches through heuristic methods [23,24]. The main goal tends to lead us towards finding an optimal way to cover a distance and smooth out the traffic, taking into account the transport costs, the social impact, and aspects regarding the clearance times or the rate of delays.

## 3. Presentation and Investigation of the Transport Structure Analysis Model

**Figure 1:**
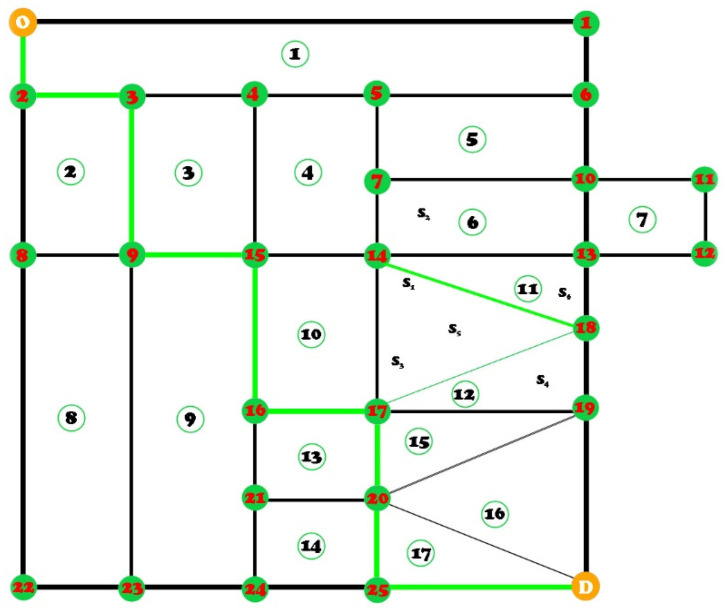
Representative diagram of a structure that optimizes the transport of large tonnage. The delimitation of the point of origin and the presentation of the destination point D, but also the division of the restricted areas into approximately 17 zones with 27 nodes capable of covering 5 km^2^.

Extremely many cities have implemented strategies by which the restriction of heavy traffic was the main goal to reduce regional environmental pollution, but also traffic congestion. In addition to these elements, it was also discovered that the studied problem also leads to the loss of the population in the severely affected areas, for obvious reasons. The involvement of the social factor and the loss of people through their reallocation to other areas define the seriousness of the problem, with extremely high costs in terms of the social factor. According to many studies and statistics conducted by inter-viewing urban residents, the presence and tolerance of oversized vehicles in residential areas is much lower than in strictly industrial areas. According to Figure 1, the transportation vehicle departs from an origin point denoted by P and arrives at a delivery destination indicated by L within the network. Within this network, there are approximately 27 nodes, and those connecting lines represent the routes. We can say that the routes are delimited in relation to the restricted area. Each zone benefits from its own properties depending on the coverage area, so when a heavy tonnage vehicle travels one of those routes, it will create a negative impact on those zones by turning them into closed routes. We can say that every truck that travels the route on the identifiers *k* and *l*, will receive the notation (*k*, *l*). If we simulate a route identified as UN, the vehicle is on the green line, and when its route passes through coordinates (U, 2), it affects zone 1, while people in zones 1 and 2 can also be affected when he takes an alternative route (2, 3). Thus, for each area, we can calculate the total loss *W*, and each route has a certain loss, as would be the case of the route (14, 18), and a total loss and a distortion of the area through the social impact can be expressed as:

(1) $$W_{14,18}=\frac{1}{2}\;\left(\frac{S_{1}\;W_{9}}{S_{1}+S_{2}+S_{3}}+\frac{S_{1}\;W_{10}}{S_{1}+S_{4}+S_{5}+S_{6}}\right),$$

where *W* represents the loss from the process when the truck travels the route on the intervals (14, 18), $W_{9}$ and $W_{10}$, and *S* is represented as the losses for those distance intervals [25].

We can assume that congestion and road activity is extremely different for each area with different time horizons. In this case, if we do not take into account the aspects of the social cost, a large vehicle must transit an area on the shortest path to obtain the most optimal route. Thus, the negative impact on traffic congestion, road and pedestrian safety in areas with a traffic density, but also its congestion are significant elements that a large vehicle can create. We can say that a truck must avoid important areas known for road problems to reduce part of the social costs. Therefore, we establish a model considering both the environmental impact and the involvement of social costs, based on an optimized model [26].

### 3.1. Discussion Regarding the Route Optimization Model for Oversize Vehicles Taking into Account the Impact of Environmental Pollution

We can say that compared to small cars, oversized vehicles transit the road sections more slowly, creating a sound discomfort, and given the large chassis and bodies, they cause congestion in many cases. According to existing studies, the conversion standard of a truck is 4 times higher than in the case of a small vehicle. Therefore, the freight transport vehicle also produces a higher amount of emissions, especially when it uses road segments with speed limits, decelerations, urban areas, and the use of lower gears. Because of the over-dimensioning, maneuverability decreases substantially, but also because of its load, sometimes causing negative and unforeseen events in traffic. Thus, the article deals as much as possible with the problem of optimizing traffic patterns to shorten the routes used by oversized vehicles to reduce the negative impact created on today’s society [27].

#### Analysis of the Costs Generated by Traffic Congestion, Referring also to the Cost of CO_2_ Emissions

Oversized vehicles dedicated to freight transport can increase the degree of traffic congestion. Because of the characteristics that such vehicles have, and here we refer to the body elements, the running speed, and the extremely heavy handling, all these elements lead to urban jams. Thus, we define VTC as the cost of traffic congestion through freight transport vehicles, defining everything as a measure to control the marginal cost in terms of congestion created by trucks traveling on public roads [28].

(2) $$VTC=\underset{i}{\sum }\underset{j}{\sum }\underset{t}{\sum }\underset{m}{\sum }\underset{k}{\sum }\lambda_{1}\;B_{m\;}T_{ij}^{t}\;N_{ijm}^{t}\;\psi_{m,k}\;x_{ij,k}^{t},$$

$$T_{ij}^{t}=t_{ij}\;1+a\left(\frac{q_{ij}^{2}}{C_{ij}}\right).$$

where $B_{m}$ represents the conversion coefficient for type m truck being later converted to a standard vehicle. $T_{ijm}^{t}$ represents the number of trucks of type m that can transit route (*i*,*j*) at time *t*. $x_{ij,k}^{t}$ represents the decision variable, and $x_{ij,k}^{t}=1$ can be interpreted as a truck of type *k* passes through the route (*i*,*j*) at the moment of type *t*, otherwise $x_{ij,k}^{t}=0$. In the case of $\psi_{m,k}$, we define it as the decision variable, $\psi_{m,k}=1$, we can say that a truck *k* belongs to the type m, otherwise $\psi_{m,k}\;$= 0, $\lambda_{1}$ is represented by the conversion factor related to traffic congestion costs. $T_{ij}^{t}$ can be identified as a function that represents the resistance of the road for the indicated route (*i*,*j*) at a given time *t*. In this work, we use functions that correspond to the existing traffic characteristics and transpose the real elements into simulation models. $T_{ij}^{t}$ can be considered the free travel time of a road on which a truck runs on route (*i*,*j*), and qt refers to the traffic flows on route (*i*,*j*) for a certain moment of time *t*. In the case of using simulation models for big data dedicated to high-tonnage transport, we can obtain real-time traffic flows directly from urban routes. $C_{ij}$ is defined as the capacity of the route presented on route (*i*,*j*), and a and b represent the parameters of the road resistance function [29].

If we refer to the different types of trucks that pollute and produce an extremely large column of emissions due to the load they have, but also the congestion that keeps them in a stationary position, all of these have a common cause. The need to classify these freight transport trucks is based on several models [30]. We can say that these oversize vehicles are divided into four categories: Heavy-Duty Gasoline Vehicles (HDGV), Light-Duty Diesel Vehicles (LDDV), Medium-Duty Diesel Trucks (LDDT), and Very Large-Duty Vehicles + 30–50 t on diesel (HDDV). These oversize vehicles have total weights exceeding 3.5 tons, and CO, NOx, PM, and hydrocarbon emissions are emitted throughout their entire operating period. In the manuscript, we try to approach the entire process of analysis and determination of these factors, approaching top models MOBILE6.7 being one of those recommended by the Environmental Protection Agencies. If we need to determine several factors, we need the analysis of the vehicle mass, later we obtain emission factors for different speeds, an aspect that can also be seen in Table 1. If we use the determination of the least squared error in order to obtain a curve of speed in relation to emissions, we can obtain emission factors for any speed range that the vehicle has. The studies carried out by other research groups and competent institutions say that they have not always taken into account the impact that pollution has on the soil. The level of tolerance of people is extremely high in conditions where in intensively exploited areas the degree of pollution is often exceeded [31]. Therefore, we propose several simulation models through which we identify solutions and present valid data, through which the tolerance index can decrease radically, in our case it is defined as *D*.

(3) $$D_{f}^{t}=U_{f}\;G_{f}^{t},$$

where $D_{f}^{t}$ represents the tolerance of the area *f* for the moment of time *t*. $U_{f}$ represents the total extent of the zone *f*, $G_{f}^{t}$ indicates the extent at the moment of time t, this being also expressed by the density of the activities of the area, related to the total cost of emissions, which are expressed as follows:

(4) $$RC=\underset{i}{\sum }\underset{j}{\sum }\underset{t}{\sum }\underset{m}{\sum }\underset{k}{\sum }\underset{w}{\sum }\lambda_{2}\;D_{f}^{t}\;N_{ijm}^{t}\;L_{ij}\;E_{ij,m}^{wt}\;\psi_{m,k}\;x_{ij,k}^{t},$$

where $L_{ij}$ is characterized as the length of the initial route (*i*,*j*), and $E_{ij,m}^{wt}$ is represented by the emission factor of the type m pollutant traveling the route (*i*,*j*), referring to the speed of the truck, $\lambda_{2}$ defines the conversion factor of the total costs that the emissions produce [32].

## 4. Implementation of the Simulation Model and Analysis of the Urban Area

We can say that the problems highlighted in the direction treated in the article can be solved with the help of heuristic algorithms, this being able to expose evolutionary simulations based on the migration behavior in search of food by ants. When they are looking for food, although they cannot communicate, each of them leaves a certain concentration of pheromones on the path they have just traveled, being a way to be found by the rest of the colony or to know the return route. Therefore, ants use this method of indirect communication to achieve empirical and often effective coordination for the whole group. If at first, the process seems chaotic and collective cooperation seems like a random movement without a specific goal, gradually the colony will stabilize and find the optimal path to the route outlined by other ants [33]. We can say that compared to other heuristic algorithms, the ACO algorithm can have an extremely large capacity through a much more branched global search, but also an extremely well distributed mechanism based on self-learning. Thus, effective pheromone-based cooperation between ants improves the ability to find the most optimal solution. We consider it important to apply the ACO algorithm in the optimization of traffic, and light signals, being ideal for intersections with more than 3–4 exits. We can say that it is the easiest way to study the performance of different ant colony algorithms. The totality of convergence rates based on pheromone concentration in the optimal path identification process is examined. Afterward, the proposed ACO algorithm is tested on an intersection/urban area with different rates and densities of vehicles, analyzing the average delay, the behavior, but also the result obtained after processing with the help of the algorithm [34]. 

If we consider a solution space, and in it each node represents a probable solution for the identified optimization problem. Thus, we can highlight several stages through which ACO is summarized, a first step is an initialization, in which the pheromone values on each node receive a constant value. The second step is the construction of the solution, each ant starts from a starting position/node, moving to another neighboring node based on the pheromone values. Mostly, ants move from node *i* to node *j* based on the following probability, also defined as the proportional rule or the ACO transition probability:

(5) $$\;\;\;p_{ij}=\{\begin{array}{c} \frac{\tau_{ij}^{\alpha }\eta_{ij}^{\beta }}{\sum_{\;\;l\in N_{\begin{array}{c} i \end{array}}}\tau_{ij}^{\alpha }\eta_{ij}^{\beta }}\;\;\;if\;j\in N_{i}\\ 0\;\;\;\;\;\;\;\;\;\;\;\;if\;j\;\notin N_{i}\;\; \end{array}$$

where $N_{i}$ represents the set of all nodes in the neighborhood of *i* that an ant has not yet visited, including all possible nodes that an ant can consider in the process of moving to node *i*. The $\tau_{ij}^{\alpha }$ represents the value of the pheromone between the nodes *i* and *j*, and *n_ij_* represents the heuristic information, available a priori, later having the possibility to calculate the reciprocal between the distances of nodes *i* and *j*, defined by $\eta_{ij}^{\beta }$. We can say that the values of *α* and *β* are usually applied dependently, they define the importance of the pheromone and the heuristic values [35]. The existence of a distinct potential from the multitude of different nodes, the probability of transition also appears, however, Equation (5) presents the basis in terms of ACO algorithms and the most used by specialized literature. The updating of the pheromone trail depends directly on the alternative paths, but also on the specific modality through which the algorithm traces the respective routes, but mostly it has a general form. If we take into account the fact that, they evaporate:

(6) $$\tau_{ij}\left(n+1\right)=\left(1-\rho \right)\;\tau_{ij}\left(n\right)$$

where *n* is the index of iteration, and ρ∈(0,1], represent the evaporation rate for pheromone. Thus, the pheromone can be on one of the updated paths or not, that’s why we use:

(7) $$\tau_{ij}\left(n+1\right)=\tau_{ij}\left(n\right)+\Delta \tau_{ij}$$

where $\Delta \tau_{ij}$, represents the pheromone update, and this is determined by another specific algorithm. Although according to the presented procedures, we build a solution based on repeated updates, ACO is successfully applied in the reconstruction and reorientation of routes [36]. We can say that ACO is applied in redundant problems, task allocation, route planning, telecommunications branching, elements, and tasks that concern traffic management systems. We consider traffic a random movement based only on the drivers’ instinct, but also on traffic signs or light signs that guide a traffic participant to a certain route, but otherwise, all other movements are completely unpredictable. We can say that in the case of the colonized optimization algorithm, an artificial ant is a calculation agent in order to identify the best solution. In the first stage, for the application of an ACO algorithm, the optimization problem must be converted to finding the shortest path from the proposed plan. Therefore, as the first step for each iteration, an ant builds a stochastic solution, following exactly the traffic nodes. Comparing all the paths found by the ants and finally updating the pheromone levels for each identified path [37].

### 4.1. Simulative Implementation of the Case Study in Relation to the Analyzed Urban Area

The area analyzed from all points of view and for which the modeling is carried out belongs to the economic-industrial perimeter of Suceava, which in the last decade has been facing extremely persistent traffic congestion. According to the studies carried out, Suceava ranks first in Romania in the number of square meters of commercial space per capita. These aspects lead to traffic congestion and a leading place in terms of the number of road accidents. Together with the acceleration of urbanization and the outsourcing of companies or their removal from urban areas, the development in the peripheries accelerated, but at the same time, they created considerable discomfort for the population. Although many years ago, the local administration departments tried some measures by which the circulation for specific categories would be limited to several alternatives, the expected results were not obtained. 

**Figure 2:**
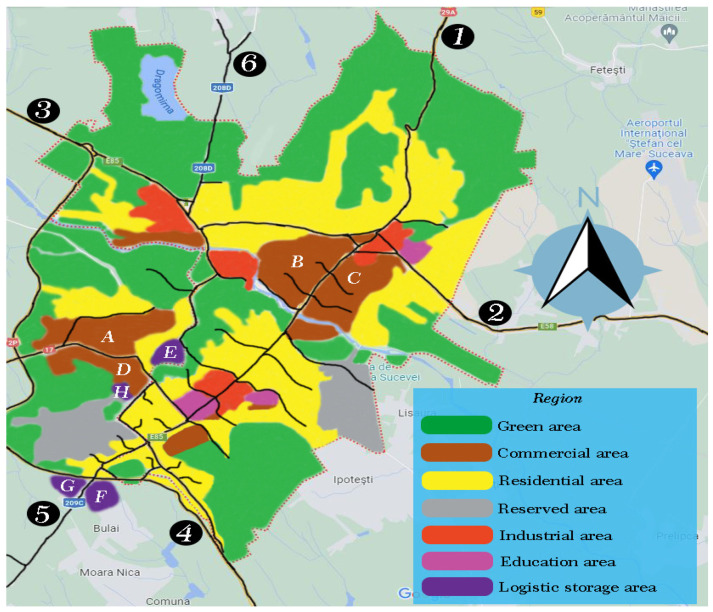
Graphic representation of the urban area and the distinct presentation of the economically and technologically developed components of Suceava. Distribution by important areas from (A–H) and the number of entrances and exits with several traffic lanes from ① to ⑥.

The current situation, altered by the border conflict between Russia and Ukraine, has recently made the traffic in the entire county lacking in mobility and safety. We consider the application of simulation models on practical cases, such as Suceava, because the reliability and applicability can be demonstrated in a practical case. Several analyzes and experiments were performed to test the effectiveness of the proposed model. Therefore, Figure 2 aims to present the economic-industrial, but also technological development area of Suceava which totals approximately 52 km^2^, which includes approximately 8 main roads and approximately 30 secondary roads, without highways or high-speed road sections. The population of the city, the county seat, is approximately 90,000 inhabitants, and the density is 1771 inhabitants/km^2^. The areas were demarcated according to the utility of the land, being represented with different colors, keeping only the main traffic lines.

**Figure 3:**
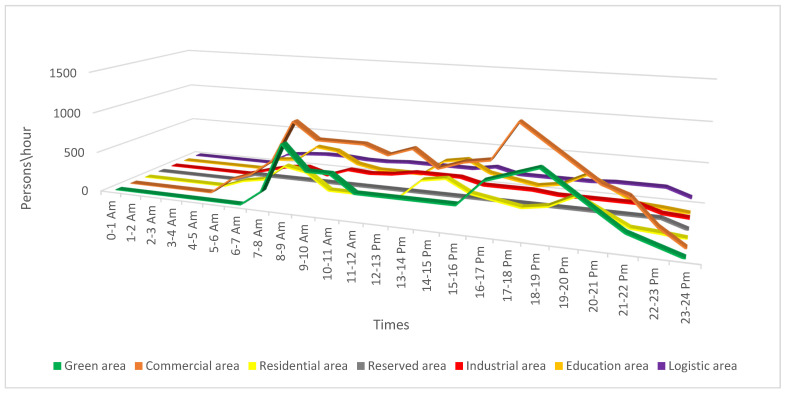
Presentation of the distribution in urban areas of the population in relation to the peak hour average.

The properties of the lands and their utility were represented by distinct colors, and the black lines are the traffic networks and internal roads for each area, adjacent streets or single-lane roads were not represented. Logistics stations for freight transportation were identified for high-density areas and approximately six entrances for the entire perimeter. Freight transport vehicles enter or leave on any numbered route from ① to ⑥. In order to simplify things, even more, traffic data and hourly distributions were analyzed according to the transit on those routes, these being presented in Figure 3, with the mention that the most transited hours and the density of vehicles in relation to what the infrastructure supports. Subsequently logistic stations for freight transportation with the dimensions of the source were implemented at a simulative level and they were presented separately in Table 2.

The design of a traffic model based on ACO algorithms becomes an extremely important premise, solving a big problem facing today’s society. Therefore, choosing a dedicated range of values between α and β to identify common nodes and subsequently obtain a conversion. Several scenarios have been outlined through which a logistic process is analyzed that refers to ACO in terms of traceability and the meticulous organization of the heavy transport process that could avoid areas with difficult circulation. The creation of automated logistic stations with large areas and the contouring of single lanes to create an environment conducive to the entry of freight transport vehicles. Therefore, freight transport vehicles enter any route presented separately, but take into account the size of the source and the density of traffic, reorganizing their route according to them.

We can see that there are no exact entry rates for the analyzed areas, and the applied model will aim to highlight exactly for durations that exceed 10–15% waiting times comparing much shorter routes. If the length of the route is greater than the imposed value, special costs and density increase, an aspect that does not create a favorable framework for the reorganization of the route. The scans are different depending on certain moments, to be able to observe the logic of the optimization of the transport ways and the way they differ from one moment to another.

We would like to mention that the data presented are based on an average of the traffic analysis, but also of the people who transit the area within a day. These data can be modified depending on the specifics of the day, especially if we are talking about weekends or holidays. Figure 3 shows the data on the distribution of the flow of people, recorded in a time interval and for a certain category of area, whether we are talking about relaxation, commercial areas, residential, education, or industries.

### 4.2. The Implementation of the Simulation Model and the Presentation of the Architecture Created in order to Obtain a Logistic Flow for the Transport of Large Tonnage

**Figure 4:**
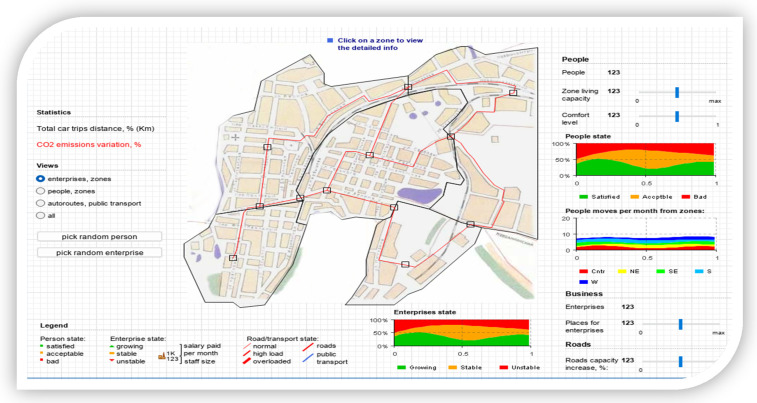
Simulative diagram showing the architecture and its functionality, as well as the routes.

In this section, the models are applied regarding data extraction from the analyzed area, considering the aspect that only sectioning of the entire plan was carried out, relying only on the areas with alternative routes and multiple access roads without being taken into account and adjacent. Therefore, we set out to analyze the simulative evolution of vehicle densities about the level of people transiting those areas. Therefore, observing in stages each movement in the front subjected to the ACO algorithm and how the random movement creates new transit routes trying to avoid the agglomeration of routes, congestion, but at the same time the degree of polluting emissions. We mention the fact that the simulations were carried out with the help of resources based on an Intel Core I9 processor, supported by o NVIDIA 2060 GPUs (Nvidia, Santa Clara, CA, USA), to highlight the convolutional process over time and the emergence of the proposal even if we expose those areas to intense traffic, increased densities, but also branched congestion. The design of the standard architecture was based on the central area with the exclusion of the bordering sequestrations without a transit that could negatively or positively influence the simulations. Thus, the defining elements of the areas were appropriately proportioned, the standard areas were delimited from those dedicated to heavy tonnage transport and later the road infrastructure was designed according to the real cases. Within the same architecture, a density factor was also taken into account, a maximum saturation of the roadways, but also aspects related to pedestrian density, all information is presented in Figure 4.

The presentation of the basic architecture is as follows, in addition to the projection of the analysis area, there are visualization modules for limited categories, such as areas, adjacent routes, public transport, transit areas, or people. Thus, we consider it imperative to customize the status of the zones and the migration of both people and vehicles according to what ACO can optimize. The gradual increase of all parameters regarding the capabilities and how these criteria are satisfied. Within the legend are also presented the elements related to the understanding of the networks created at the level of the simulation stage, the lines with a high contrast following the measurements, represent the guidelines for the delimitation of the respective areas. Zone logical stations provide insight that can pencil new exits to other alternative routes suggested using ACO algorithms to establish a new logical connection at the node level. Establishing a new network-wide communication rate and allowing a direct connection between the source and the other vehicles in the network. The need to present an accurate distribution of travel or transit time for different areas according to the time interval is not negligible, but at the same time, it is not a constant in terms of daily activities. This however provides a viable perspective by folding with the ACO algorithm through the prism of random movement and finding paths out of the network of nodes. We can say that as the tests progress, the commercial areas are distinct from the other areas and later transit alternatives can be distinguished from the industrial or residential areas. Aspects regarding the social costs in terms of peak hours represent another challenge because they have extremely high increases through the prism of the network distribution in the travel rates within the spatiotemporal network.

**Figure 5:**
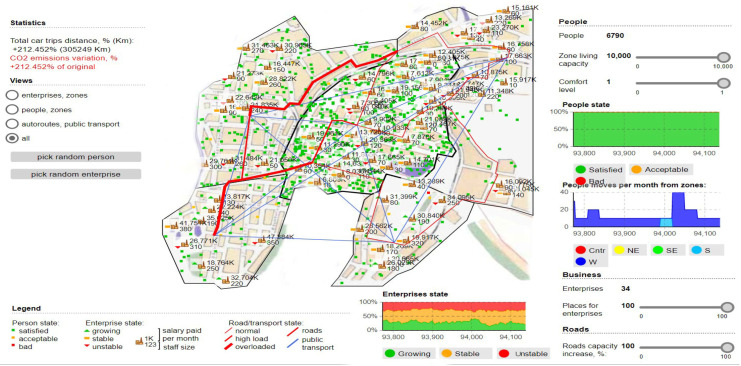
Simulative scenario in which the ACO model are presented in relation to traffic densities and transit capacity in relation to the capacity of areas and nodes through which transportation vehicles can pass.

**Figure 6:**
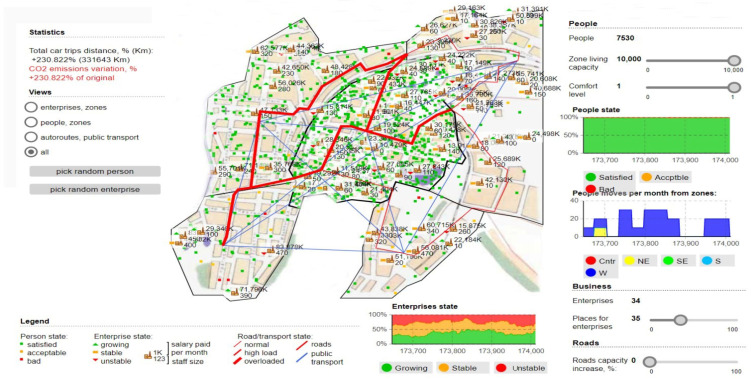
Simultaneous scenario showing the instability of transit routes and the degree of congestion created, as well as CO_2_ emissions.

Therefore, there are aspects and phenomena through which freight transport costs are considerably reduced, but to a large extent, other branches are also affected, including social costs. Taking these aspects into account, the model also aims to analyze the severity, optimizing freight transport operations and finding the shortest method that would not directly impact the environment. To highlight this aspect in the simulations, percentages will also be presented with how the polluting emissions are present at the given time and how it is about normal exposure without the use of a dedicated fluidization algorithm, see Figure 5 and Figure 6.

Figure 5 shows the results of the simulations, raw data from the transport stations, the distribution of time about the analyzed areas, and how the transit of the perimeter is affected according to the traffic flow. Only the simulations showing a significant difference influencing traffic density and congestion negatively were extracted.

**Figure 7:**
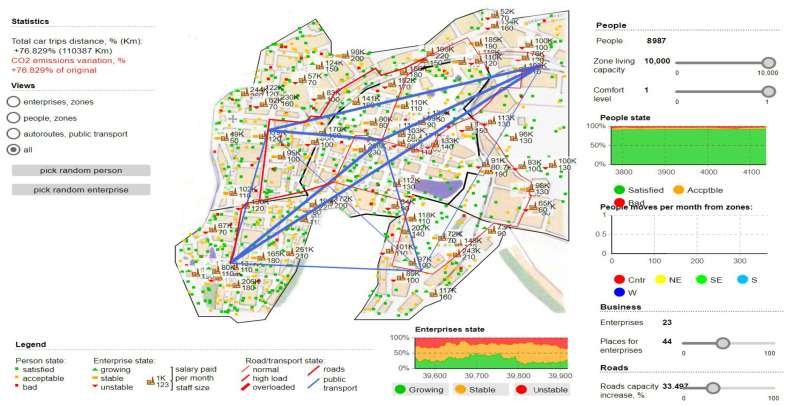
Demonstration scenario showing the contribution brought by the ACO model to the transport network, the exposure of the solutions generated by the algorithm and the distinct highlighting of the new transit areas with the aim of streamlining traffic and reducing polluting emissions.

Regarding the validation of the model and the impact it has on urban areas, we can see how the areas that become congested are distinctly outlined and at the same time the degree of satisfaction of the population and the way it is affected by oversized transport. We can see that to obtain a maximum degree of analysis, comfort zones were established and the model was applied to all areas, including public transport, commercial or industrial areas. The commercial environment and business area aspects have a downward slope, and the most massive impact the ACO algorithm has had is fading path lengths by up to 8% compared to standard path rejection factors exceeding 30 % of the coverage area. By further analyzing and imposing a logistics process through which truck transport routes have significant changes. Thus, we observe the existence of new time horizons in the contouring of the access paths dedicated to the logical regression measures constantly applied to the change of the analysis areas. In Figure 7, the application of the ACO algorithm is fully implemented, observing the most important characteristic, which is the one through which the degree of CO_2_ emissions decreases considerably. The simulation was performed in tandem with the maximum degree of occupation of the habitable and passable area, and the degree of satisfaction and comfort of the population being affected. We can see that the total distance traveled is clearly superior to the other routes and that transport vehicles are moved away from high-congestion areas to other routes. These routes have been mapped distinctly from previous simulations, although the random movement of densities is in the same viable nodes that have been used to increase the performance of the transport network. We can say that the most important results pertain to the analysis of the longest lengths and in the case of peak times, the ACO alternatives seem much more feasible from all points of view, exceeding the expectations outlined in this regard.

The in-depth analysis of this simulation dedicated to optimization paths for logistic transport materialized through aspects related to how they affects the social level. It was found that the level of emissions and the degree of congestion are not maintained when applying a distribution to the level of the travel rate in the spatio-temporal network. Therefore, the phenomenon of reducing the density of logistic traffic decreases social costs increases the degree of comfort, and at the same time has a positive impact on traffic safety. The complexity of the proposed model has increased traffic efficiency by more than 33%, the distance between the nodes in the traffic network remains the same, only that new, significant time horizons are outlined, and transit hours are exposed in a condensed manner. We can say that the model in its current state still needs adjustments to restrict routes according to the areas allowed for transit by freight transport.

## 5. Conclusions

The case study was concentrated on the industrial and commercial area of the city of Suceava because the validity of the proposed model must demonstrate its viability in a practical case. In this work, three scenarios out of more than 15 were selected that highlight the progress that the proposed model brings especially the shortest path and the proportions of polluting emissions. We can say that the travel lengths between the nodes in the route have been optimized accordingly and that the impact on the environment can be observed, showing a consistent improvement, matching the density of urban traffic. The ACO model can reduce congestion in an extremely efficient way, thus polluting emissions of vehicles dedicated to transport are reduced, even if the distance between them and nodes has increased. Therefore, the results show that traffic congestion is one of the most important elements that we have to deal with regardless of the field in which we operate, and emissions greatly influence social comfort. The algorithm has the feature of improving travelers’ safety and working in social welfare. The simulation model also presented a difference between the shorter paths and the distribution of the travel rate within the spatio-temporal network, characteristics in which the route was optimized taking into account the transit hours, experimental data being exposed in the entire interval (24:00 p.m.–06:00 a.m.), but also peak hours (10:00 a.m.–16:00 p.m.). We can guarantee the viability of the solution to be implemented at the level of traffic management infrastructures and the adoption of new control strategies. The research provides not only a quantitative basis for the development of simulation models but also the solution to be able to help drivers and society towards optimal paths in real-time. Bringing new theoretical guidelines and placing logical stations that would plan traffic, according to the type of vehicles and their density would be a viable solution. Combining simulation models with practical implementations, based on emerging technologies capable of providing information in real-time becomes a certainty through technological mixes and communications even through visible light [38]. These applications can be found in road safety systems, pedestrians, and autonomous vehicles. Therefore, the main goal of all studies is to reduce the number of deaths caused by traffic and streamline or eliminate traffic congestion. We can say that a main future direction is the one in which intelligent traffic fluidization and monitoring systems will be implemented based on communication protocols capable of communicating with the infrastructure or vehicles (V2X)–(V2I). These systems will incorporate both the part of simulative models transposed into basic principles through which the decision-making part will work in the mentioned direction.

## Figures and Tables

**Table 1:** Exposure of noxious emission factors according to speed (g/km).

Speed Vehicles	Emissions	Heavy-Duty Gasoline Vehicles	Light-Duty Diesel Vehicles	Medium-Duty Diesel Trucks	Very Large-Duty Vehicles
5	CO NOx	68.12 2.56	27.44 25.51	29.65 27.33	32.53 32.79
10	CO NOx	58.22 2.66	21.38 24.78	22.45 24.72	28.44 30.70
15	CO NOx	44.51 2.70	17.31 21.44	20.28 22.15	26.51 28.83
20	CO NOx	40.33 2.79	20.87 15.21	23.67 18.12	25.79 24.65
25	CO NOx	39.74 2.83	19.54 16.65	21.81 16.21	22.74 21.07
30	CO NOx	38.33 2.91	12.17 10.22	20.78 14.41	20.49 18.91
35	CO NOx	37.01 3.07	11.77 13.52	12.83 11.49	19.01 17.38
40	CO NOx	40.14 3.28	8.91 12.49	11.14 10.51	18.83 18.49
45	CO NOx	41.66 3.45	7.26 11.84	10.84 9.91	16.02 19.28
50	CO NOx	43.59 3.73	6.29 10.93	9.46 9.22	10.45 17.73

**Table 2:** Presentation of the arrival rates of freight transport vehicles in each logical station.

Arrival Time (veh/h)	①	②	③	④	⑤	⑥
A	30	28	20	17	10	9
B	18	22	21	16	19	11
C	22	33	11	19	24	13
D	17	21	9	22	17	23
E	14	9	11	16	9	17
F	7	15	13	21	8	12
G	12	19	8	4	5	4
H	9	5	4	2	2	7
